# CRISPR/Cas9-Mediated Knockout of the *Corazonin* Gene Indicates Its Regulation on the Cuticle Development of Desert Locusts (*Schistocerca gregaria*)

**DOI:** 10.3390/insects16070704

**Published:** 2025-07-09

**Authors:** Yingying He, Qiang Yan, Yong Bi, Guosheng Liu, Shuang Hou, Xinyi Chen, Xiaoming Zhao, Xueyao Zhang, Min Zhang, Jianzhen Zhang, Binbin Ma, Benjamin Warren, Siegfried Roth, Tingting Zhang

**Affiliations:** 1 Research Institute of Applied Biology, Shanxi University, Taiyuan 030006, China; heyingyinging@163.com (Y.H.); yongbi1998@163.com (Y.B.); zxming@sxu.edu.cn (X.Z.); zxy@sxu.edu.cn (X.Z.); minzhang@sxu.edu.cn (M.Z.); zjz@sxu.edu.cn (J.Z.); 2 School of Life Science, Shanxi University, Taiyuan 030006, China; 3 Institute of Zoology-Developmental Biology, University of Cologne, 50923 Cologne, Germany; 4 State Key Laboratory of Cotton Bio-Breeding and Integrated Utilization, School of Life Sciences, Henan University, Kaifeng 475004, China; yanqiang@henu.edu.cn (Q.Y.); lgslgsh@126.com (G.L.); hazelhou@yeah.net (S.H.); 5 Guangdong Provincial Key Laboratory of Insect Developmental Biology and Applied Technology, Guangzhou Key Laboratory of Insect Development Regulation and Application Research, Institute of Insect Science and Technology, School of Life Sciences, South China Normal University, Guangzhou 510631, China; 2024010247@m.scnu.edu.cn; 6 Department of Biology, Johns Hopkins University, Baltimore, MD 21218, USA; bma13@jhu.edu; 7 School of Life Sciences, Keele University, Huxley Building, Newcastle ST5 5BG, UK; b.warren@keele.ac.uk

**Keywords:** CRISPR/Cas9, corazonin, *Schistocerca gregaria*, cuticle

## Abstract

The desert locust (*Schistocerca gregaria*) represents one of the most destructive agricultural pests globally, renowned for its ability to form massive swarms that can devastate crops and threaten food security across vast regions. Despite the widespread application of the CRISPR/Cas9 gene-editing system in several insect orders, its utilization in locusts, particularly in the desert locust, has remained relatively unexplored. We established a CRISPR/Cas9-mediated gene-editing workflow for the desert locust using gene encoding for neuropeptide corazonin (Crz) as a target. We also analyzed the phenotypic and physiological characteristics of the mutant using paraffin sectioning, HE staining, and chitin staining techniques. Our findings revealed that while *Crz* knockout desert locusts were viable and maintained normal fertility, they exhibited striking phenotypic alterations, including albinism and a significant reduction in cuticle thickness. These observations not only highlight the functional role of Crz in pigmentation and cuticle development but also underscore the potential of CRISPR/Cas9 as a powerful tool for dissecting gene function in locusts. Furthermore, the successful application of CRISPR/Cas9 in desert locusts also paves the way for similar genetic studies in other non-model insects, expanding the scope of functional genomics in entomology.

## Simple Summary

The desert locust is a highly destructive agricultural pest that forms massive swarms that devastate crops and trigger severe food shortages in affected regions. Although the CRISPR/Cas9 gene-editing system has been widely applied in various insects, its use in locusts remains limited, particularly in the desert locust, *Schistocerca gregaria*. In this study, we systematically established a CRISPR/Cas9-mediated gene-editing workflow for desert locusts by targeting the encoding gene of corazonin (Crz). We analyzed the effects of *Crz* knockout and found that while Crz-deficient desert locusts were viable and fertile, they exhibited an albino phenotype and reduced cuticle thickness. These results provide new insights into the function of Crz in locusts and lay a foundation for further gene-editing research in desert locusts.

## 1. Introduction

The desert locust (*Schistocerca gregaria*) is one of the most notorious agricultural pests worldwide, capable of forming massive swarms that devastate crops and threaten food security [1,2,3]. Understanding the genetic mechanisms underlying locust biology is highly beneficial for developing new pest control strategies. However, due to their large genome and long reproductive cycle, genetic studies in desert locusts have been challenging.

The CRISPR/Cas9 system has revolutionized genetic research across various organisms, including insects, and has become one of the most efficient and easy-to-use technologies among existing methods [4,5,6,7,8,9]. CRISPR/Cas9-mediated gene-editing technology has been extensively applied in disease prevention and treatment, livestock genetic enhancement, crop improvement and gene function research [10,11,12,13,14]. While CRISPR-based gene editing has been widely implemented in model insects such as *Drosophila melanogaster*, *Bombyx mori*, and *Aedes aegypti*, its application in locusts remains relatively limited [15,16,17,18]. In *Locusta migratoria*, CRISPR/Cas9 has been successfully employed to investigate genes involved in swarming behavior and pigmentation [19]. However, studies using CRISPR in *S. gregaria* remain scarce, limiting the potential for genetic research in this genus. Earlier research on *S. gregaria* used conventional Cas9 to edit the *SNMP1* gene, but the lack of a standardized gene editing workflow hindered its applicability [20].

Corazonin (Crz) is a highly conserved neuropeptide in insects that plays a crucial role in regulating fundamental physiological processes [21,22]. In *D. melanogaster*, the Crz neuropeptide acts as a stress hormone and regulates stress-related behaviors and metabolism [23,24]. In *Blattodea*, Crz has been identified as a potent cardioaccelerator [25]. In *B. mori*, Crz may play a role in regulating larval growth and the rate of silk spinning [26]. In *Anopheles gambiae* and *Rhodnius prolixus*, this neuropeptide has a significant role in regulating heart physiology [27,28]. In *L. migratoria*, RNA interference (RNAi)-mediated knockdown of *Crz* induces albinism while maintaining normal development and reproduction [29]. Subsequent CRISPR/xCas9-mediated gene-editing confirmed the critical role of Crz in melanin synthesis for dark pigmentation and cuticle development [19]. In *S. gregaria*, the function of Crz in body color regulation and phase polyphenism has also been elucidated using RNAi technology [30]. The visible pigmentation alterations caused by *Crz* mutations enable non-invasive phenotypic screening, thus making this neuropeptide an optimal target for developing precision gene-editing tools in locust research and pest control applications.

In this study, we established a CRISPR/Cas9-mediated gene-editing system in desert locusts by targeting the *Crz* gene, which provided crucial technical references and methodological foundations for developing gene-editing platforms in other species. We evaluated the phenotypic consequences of *Crz* knockout and observed an albino phenotype and reduced cuticle thickness in mutant individuals. This study presents two innovative contributions: first, the methodological advancements in a systematical CRISPR/Cas9 gene-editing system in desert locusts not only overcome species-specific challenges but also provide a generalizable technical framework for gene editing in other non-model orthopteran pests; secondly, the demonstration of evolutionarily conserved role of Crz in cuticle development among orthopteran genera offers novel theoretical insights into the molecular mechanisms underlying arthropod exoskeleton formation.

## 2. Materials and Methods

### 2.1. Multi-Sequence Alignment and Phylogenetic Tree Construction

The pro-corazonin amino acid sequences of arthropods were retrieved from the NCBI database. A total of 7 arachnid, 16 crustacean, and 36 hexapod Crz sequences were compared using MEGA7, and a phylogenetic tree was subsequently constructed. The tree was then enhanced using the online tool iTOL (https://itol.embl.de/login.cgi?logout=1, accessed on 12 November 2024) for visualization.

### 2.2. Genome Structure Analysis and Target Site Design of SgCrz

The CDS of the *S. gregaria* prepro-corazonin (LC031859.1) was used to identify the *Crz* gene in the released genome (JAKZLS010000003.1). According to the obtained *SgCrz* genomic DNA sequence, the first exon of the *SgCrz* gene was used as the targeted fragment for sgRNA design using the CPISPR-P 2.0 tool (http://crispr.hzau.edu.cn/cgi-bin/CRISPR2/SCORE, accessed on 13 May 2024). The online software of the website screened potential sgRNA target sites, considering off-target effects and other factors, and then selected the sequence with the highest score and fewest off-target sites as the target sequence.

### 2.3. Animals

*S. gregaria* adults were purchased from Kalthoffs Zoologia (Cologne, Germany) and reared in plastic cages (25 cm × 25 cm × 25 cm) in University of Cologne under controlled conditions: a temperature of 33 °C, a relative humidity of 30%, and a 16L: 8D light cycle. A sand table was placed in the cage to facilitate egg-laying. Once the eggs were laid, the sand table was removed for egg collection.

### 2.4. Synthesis and Verification of the sgRNA In Vitro

The sgRNA was synthesized according to the protocol provided in the Precision gRNA Synthesis Kit (A29377, Invitrogen, Waltham, MA, USA). The genomic fragment containing the target site was amplified with the forward primer (5′-AGTGAACACCTTTGCCTCGT) and the reverse primer (5′-TGACGCTCCCCAAGAAAGTG). PCR reaction conditions were as follows: initial denaturation at 94 °C for 5 min, followed by 35 cycles of 94 °C for 30 s, 64 °C for 30 s and 72 °C for 40 s, with a final extension at 72 °C for 10 min. A total of 300 ng of target fragment, sgRNA, and Cas9 (A36498, Invitrogen, Waltham, MA, USA) were added into a reaction tube, along with 1 μL of 10× Cas9 Buffer. Then, the reaction volume was adjusted to 10 μL with RNase-free water and incubated at 37 °C for 2 h. Agarose gel electrophoresis was performed to assess the efficiency of sgRNA [31].

### 2.5. Microinjection

Microinjection was performed as previously reported [31]. Briefly, fertilized eggs were collected within 2 h after spawning, washed and arranged on injection plates. Amounts of 1 μL of sgRNA (300 ng/μL), 1 μL of Cas9 (300 ng/μL), and 8 μL of RNase-free water were mixed for injection. Desert locust eggs were injected using the microinjection system (TransferMan 4r, Eppendorf, Hamburg, Germany), with an injection volume of approximately 30 nL per egg. The injection parameters were as follows: 200 hPa of the injection pressure (pi), 0.5 s of the injection time (ti), and 10 hPa of the compensation pressure (pc). Injected eggs were transferred to a Petri dish lined with moistened filter paper and placed into a 33 °C incubator.

### 2.6. Mutant Screening and Homozygous Mutant Acquisition

Genomic DNA was extracted from randomly selected embryos for PCR-mediated genotyping during the embryonic stage, while the remaining embryos were cultured until they hatched. Both hatching rate and mutation rate were recorded. Since Crz deficiency results in an albino phenotype, the G_1_ generation was obtained by selecting albino desert locusts from the G_0_ generation and crossing them with wild-type individuals. The G_1_ generation was screened by Sanger sequencing and peak diagram analysis. Individuals with the same mutation type were crossed to obtain homozygous G_2_ individuals. Based on the sequencing results, the online tool SMART (https://smart.embl.de/, accessed on 5 November 2024) was used to predict protein sequence changes.

### 2.7. Off-Target Analysis

The released genome was downloaded from the NCBI database. Potential off-target sites were predicted using the target-and-off-target mode of CasOT to assess the specificity of CRISPR/Cas9-mediated editing in *S. gregaria* [19]. Primers were designed to amplify the genomic fragments harboring potential off-target sites, and off-target efficiency was analyzed by sequencing.

### 2.8. Analysis of Development Duration and Fertility

Twenty mutant and twenty wild-type desert locusts were selected for observation. Their developmental progression was monitored and recorded at 12 h intervals. Each molting event marked the transition to the next instar stage, and the duration of each instar was systematically documented. After eclosion, 15 pairs of wild-type and *SgCrz^−/−^* mutants were used to estimate their adult lifespans. Four sets of wild-type and mutant desert locusts were paired, respectively, oocysts were collected every two days, and the number for each group was recorded over two weeks to assess reproductive output.

### 2.9. Hematoxylin and Eosin (H&E) Staining

The second to third abdominal segments of a four-day old adult were placed in 4% paraformaldehyde and fixed for one week, and then gradient dehydration was performed. After dehydration, the tissue was paraffin-embedded, and then the sample was cut into 5 μm-thick tissue using a microtome. The sections were placed in xylene for deparaffinization, and followed by gradient alcohol rehydration. The sections were washed with distilled water and stained with hematoxylin solution for 10 min. After washing again with distilled water, the sections are placed in the eosin staining solution for 3 min, followed by gradient alcohol for dehydration and mounted with neutral resin for subsequent observation. After the HE results were scanned, the cuticle thickness was determined using a ruler in the CaseViewer software (Version 2.4), and five samples were taken from each group for statistics.

### 2.10. Chitin Staining

Paraffin sections were first deparaffinized and rehydrated. After washing with PBS, dye with Fluorescent Brightener 28 stain solution (100 μg/μL, 910090, Sigma-Aldrich, Shanghai, China) at room temperature for 2 min. After washing with PBS again, nuclear staining was performed with SYTOX™ Green nucleic acid stain (1:5000 dilution, S7020, Invitrogen, Waltham, MA, USA) at room temperature for 15 min. Then, samples were washed with PBS. Finally, Antifade Mounting Medium (P0128M, Beyotime, Shanghai, China) was applied dropwise, and the samples were covered with a coverslip and stored in the dark.

### 2.11. Image Acquisition and Statistical Analysis

Images were captured with a scanner (EPSON PERFECTION V700 PHOTO, Suwa, Japan). Statistical significances were analyzed by the *t*-test using SPSS software (Version R27.0.1.0). The values were expressed as mean ± SD, and *p* < 0.05 considered the statistically significant. n.s., *p* > 0.05; *, *p* ≤ 0.05; **, *p* ≤ 0.01; ***, *p* ≤ 0.001.

## 3. Results

### 3.1. Phylogenetic Analysis and sgRNA Target Design

**Figure 1:**
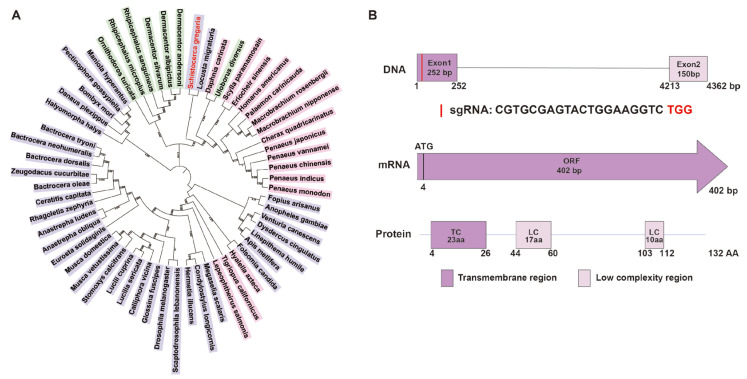
Phylogenetic evolution and gene structure analysis of *SgCrz*. (**A**) The maximum likelihood tree was constructed using the amino acid sequences of 59 pro-corazonin orthologous genes. In this phylogenetic tree, different colors represent different taxonomic groups: green indicates arachnida; pink indicates crustaceans; purple indicates hexapods. (**B**) Putative structure of *SgCrz* and sgRNA target site of desert locust. In the protein structure, the deep purple rectangle represents the transmembrane region and the lavender rectangle represents the low complexity region.

A comprehensive phylogenetic analysis was conducted on 59 pro-corazonin amino acid sequences from 7 arachnids, 16 crustaceans, and 36 hexapods to elucidate the evolutionary relationships of the *Crz* gene across various arthropod classes. The clustering of locusts and desert locusts indicates a close evolutionary relationship and suggests potential functional similarities (Figure 1A).

According to the nucleotide BLAST (https://blast.ncbi.nlm.nih.gov/Blast.cgi, accessed on 13 May 2024) analysis result, the *SgCrz* gene is located on chromosome 3 and comprises two exons. The encoded prepro-SgCrz consists of 132 amino acids, featuring a transmembrane domain and two low-complexity regions. For targeted gene editing, the target site was selected within the first exon (Figure 1B).

### 3.2. The Workflow of Gene Editing in Desert Locust

**Figure 2:**
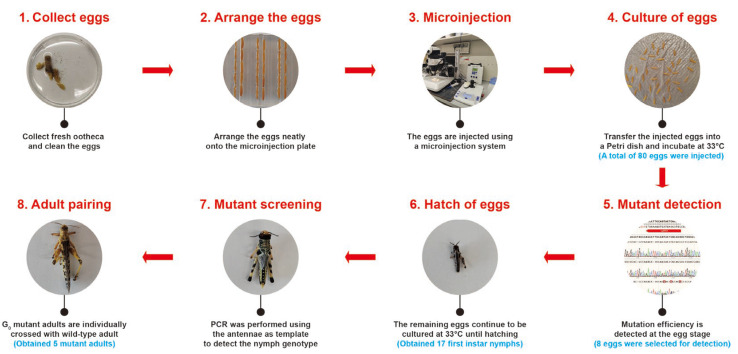
Generating CRISPR/Cas9 mutants in desert locust. There are five steps to obtain the mutant: (**1**) fresh ootheca were collected within 2 h post-oviposition, isolate the ootheca, and thoroughly wash egg with distilled water; (**2**) eggs were aligned on the microinjection plate; (**3**) the RNP complex was injected into the eggs using a microinjection system; (**4**) injected eggs were placed on moist filter paper in a Petri dish and incubated at 33 °C until hatching; (**5**) several injected eggs were randomly selected for mutation efficiency detection at the egg stage; (**6**) the remaining eggs were cultured until hatching; (**7**) the nymphs were reared in the incubator until they reached the fifth instar, after which Sanger sequencing was utilized to screen for mutants; (**8**) the adult mutants of the G_0_ were paired with the virgin wild-type. The blue font below each step represents the number of individuals for that step in this article.

To generate *SgCrz* gene knockout mutants, we used a systematic embryonic microinjection and culture protocol, comprising the following stages: (1) Collect eggs: Fresh oothecas were collected within 2 h post-oviposition, with care taken to avoid damage during handling; (2) Arrange the eggs: The eggs were cleaned and neatly arranged on the microinjection plate; (3) Microinjection: A mixture of sgRNA and Cas9 (both diluted to 300 ng/μL) was injected into the eggs using a microinjection system, and the needle was monitored continuously during injection to prevent clogging and ensure successful delivery of the solution into the eggs; (4) Culture of eggs: The injected eggs were gently transferred to a Petri dish covered with moist filter paper and incubated at 33 °C, dead eggs were promptly removed during cultivation to prevent contamination; (5) Mutant detection: After 3 days of cultivation, randomly selected several injected eggs were used as genomic templates. Target DNA fragments were amplified using the primers specified in Section 2.4, followed by sequencing to determine the mutation efficiency at the egg stage; (6) Hatching of eggs: The remaining eggs were continuously cultured at 33 °C until they hatched the first instar nymphs; (7) Mutant screening: The nymphs were continuously cultured at 33 °C until they reached the fifth instar. At this stage, using the antennae DNA of nymph as template, target fragments were amplified and subjected to Sanger sequencing for mutant identification; (8) Adult paring: Each G_0_ mosaic mutants was paired with the virgin wild-type adults in separate plastic cups to establish independent G_1_ lineages. This systematic approach ensures efficient gene editing and accurate mutation identification (Figure 2).

### 3.3. G_0_ Mutant Screening

**Figure 3:**
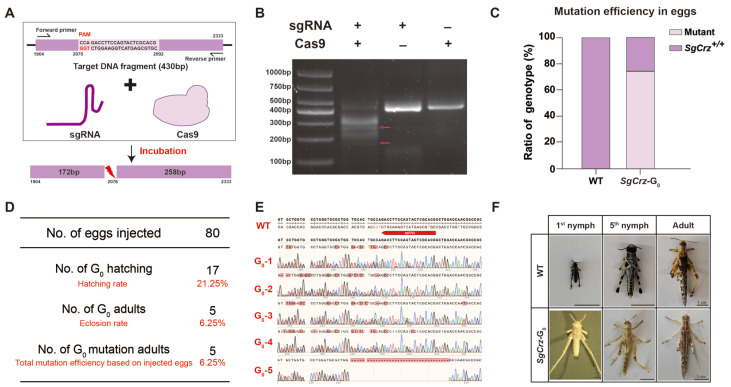
The acquisition of *SgCrz* mutant of G_0_. (**A**) Schematic of in vitro digestion of target DNA fragment. (**B**) The verification of the sgRNA in vitro. The mixture of sgRNA, Cas9 and the target fragment was incubated at 37 °C, and the enzyme digestion efficiency was detected by electrophoresis. (**C**) G_0_ egg stage mutation efficiency. (**D**) Survival rate and mutation efficiency analysis. (**E**) *SgCrz* target site sequences from G_0_ adults. (**F**) Color change of G_0_ mutant. After Cas9/sgRNA injection, the desert locust body color turned white.

At each stage of the gene editing process, we systematically evaluated sgRNA efficiency. Prior to microinjection, the target DNA fragment containing the sgRNA binding site was amplified in vitro to verify sgRNA digestion efficiency. If the sgRNA is functional, the target DNA fragment should be cleaved into two fragments (172 bp and 258 bp) (Figure 3A). Gel electrophoresis results demonstrated successful cleavage of the target DNA fragment in the presence of both sgRNA and Cas9, whereas control groups lacking either component did not exhibit cleavage (Figure 3B). Then, we microinjected RNP complexes into 80 locust eggs. Among four randomly selected samples analyzed during the egg stage, three exhibited detectable mutations, yielding an egg stage mutation rate of 75% (Figure 3C, the sequencing results are shown in Figure S1). The remaining eggs were cultured, yielding 17 first instar nymphs, corresponding to a G_0_ hatching rate of 21.25%. Five of the surviving nymphs matured into adults, all of which exhibited mutations, achieving a 6.25% mutation efficiency in total based on injected eggs (Figure 3D). The G_0_ adult exhibited a female-to-male ratio of 3:2. Sequencing analysis confirmed the introduction of mutations at the target sites. Comparison between wild-type and G_0_ locusts revealed multiple base mutations, validating the effectiveness of the sgRNA design and CRISPR/Cas9-mediated targeting (Figure 3E). Notably, G_0_ mutants displayed an albino phenotype compared to the wild-type, indicating the impact of targeted gene disruption (Figure 3F).

### 3.4. Establishment of Homozygous SgCrz^−/−^ Mutant Line

**Figure 4:**
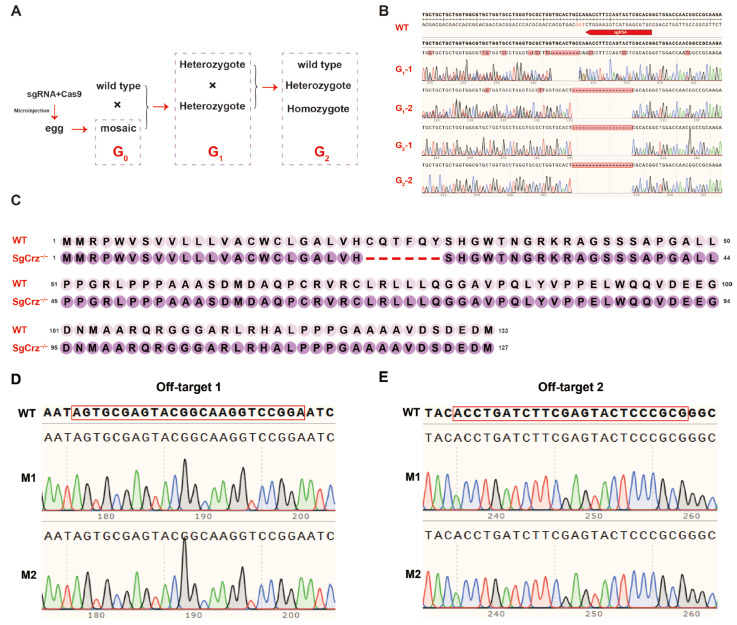
Construction of homozygous mutants. (**A**) Establishment of homozygous mutants. First, G_0_ was crossed with wild-type desert locust to obtain G_1_. After the mutation type of G_1_ was determined by DNA sequencing, heterozygotes with the same mutation type were hybridized to obtain G_2_ homozygous mutants. (**B**) G_1_ and G_2_ *SgCrz* target site mutation sequences. (**C**) Protein sequence changes after *SgCrz* deletion mutation. (**D**) Potential off-target site 1 has no mutation. (**E**) Potential off-target site 2 has no mutation.

To establish a homozygous *SgCrz^−/−^* mutant line, G_0_ mosaic individuals were crossed with wild-type locusts to generate G_1_ heterozygous mutants. The G_1_ individuals were screened via Sanger sequencing to identify those carrying the same mutation type. G_1_ individuals with the same mutations were interbred to obtain G_2_ homozygous mutants (Figure 4A). Peak maps of target gene Sanger sequencing results from wild-type, G_1_ and G_2_ individuals confirmed that the gene editing was successful. There were different number of base deletions in the *SgCrz* gene of the G_1_ mutant, and a G_2_ homozygous mutant with 18bp deletion in the *SgCrz* gene was obtained after interbreeding individuals with the same mutation type (Figure 4B). Amino acid sequence analysis revealed that the mutants had deletions at the target sites; however, subsequent translation was unaffected, suggesting that the gene function might not be completely lost (Figure 4C). To verify the specificity of the CRISPR/Cas9 editing system, two potential off-target sites were examined. Sequencing results of wild-type locusts and *SgCrz^−/−^* mutants showed no mutation at these off-target sites, indicating high specificity of CRISPR/Cas9-mediated targeting in this context (Figure 4D,E).

**Figure 5:**
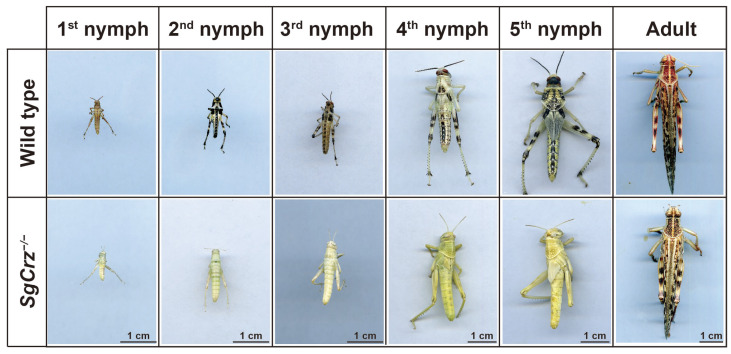
Phenotype of homozygous mutants and control for off-target mutations. Homozygous *SgCrz* mutant body color phenotype.

Morphological observations across different developmental stages (1st-5th instar nymphs and adults) indicated that *SgCrz^−/−^* mutants exhibited significant differences compared to wild-type individuals. Notably, the mutants exhibited a completely albino phenotype, implying that *SgCrz* gene knockout significantly impacts desert locust pigmentation (Figure 5).

### 3.5. Effects of SgCrz Knockout on the Life-Span and Reproductive Traits

**Figure 6:**
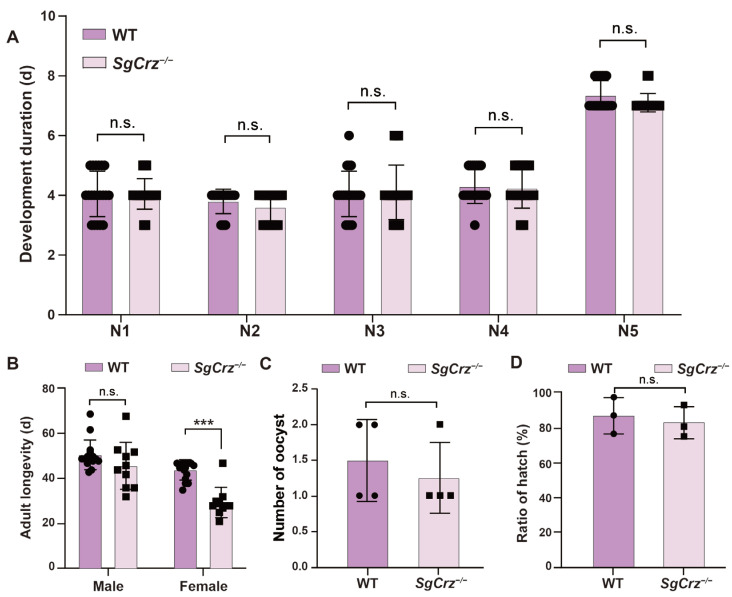
Development, longevity and fecundity of *SgCrz* mutant locusts. (**A**) Comparison of developmental duration from the first to the fifth instar between wild-type and *SgCrz^−/−^* desert locusts. n = 20. (**B**) Comparison of the adult longevity between wild-type and *SgCrz^−/−^* desert locusts. WT: n = 15; *SgCrz^−/−^*: n = 10. (**C**) Comparison of the number of ootheca number between wild-type and *SgCrz^−/−^* desert locusts. n = 4. (**D**) Comparison of hatching rates of offspring of wild-type and *SgCrz^−/−^* desert locusts. n.s., *p* > 0.05; ***, *p* ≤ 0.001.

To investigate the effects of *SgCrz* mutations on the desert locust development, the developmental duration, adult lifespan, egg-laying capacity and hatching rate of wild-type and *SgCrz^−/−^* mutant were recorded and compared. There were no significant differences in the developmental duration between wild-type and mutant locusts, suggesting that *SgCrz* mutations do not affect the duration of each developmental stage (Figure 6A). Adult lifespan analysis showed no significant difference between wild-type and *SgCrz^−/−^* in males. However, lifespan was significantly reduced in *SgCrz^−/−^* females, suggesting that the effect of this mutation on lifespan might be sex-specific (Figure 6B). There were no significant differences in the reproductive capacity and hatching rate of *SgCrz^−/−^* mutants compared with wild-type (Figure 6C,D).

### 3.6. SgCrz^−/−^ Mutants Exhibited Compact but Thinner Cuticles

**Figure 7:**
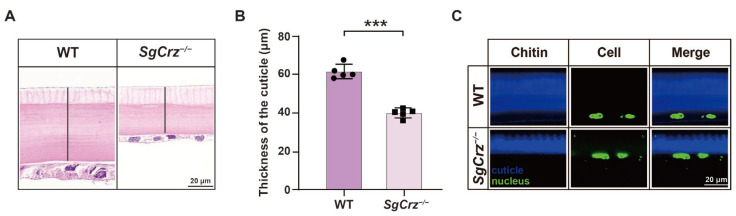
Cuticle structure of *SgCrz^−/−^* mutant. (**A**) H&E staining on the cuticle of wild-type and desert locust mutants. The black line indicates the thickness of the cuticle. (**B**) The cuticle thickness of desert locust mutants was significantly lower than that of wild-type. (**C**) Chitin staining on the cuticle of wild-type and desert locust mutants. Blue represents chitin and green represents the nucleus. ***, *p* ≤ 0.001.

Beyond pigmentation changes, we examined the effects of *SgCrz* loss on the cuticle of desert locusts. The insect cuticle is typically divided into the epicuticle and procuticle. The procuticle forms the predominant portion of the insect cuticle, primarily composed of a chitin-protein composite matrix. H&E staining results revealed a significant reduction in cuticle thickness in *SgCrz^−/−^* mutants compared to the wild-type (Figure 7A,B). Chitin staining further confirmed the reduction in chitin in the cuticle of *SgCrz^−/−^* mutants (Figure 7C). These findings suggest that the *SgCrz* gene plays a crucial role in maintaining cuticle structure in desert locusts.

## 4. Discussion

CRISPR is a new gene editing tool with great development potential, which is widely used in various fields because it is rapid, simple and efficient [32,33,34]. Practitioners in the life sciences continue to explore, optimize, and iterate existing CRISPR editing technology and expand its application [35,36,37,38]. CRISPR/Cas9 has been widely applied in *L. migratoria* for studying genes involved in swarming behavior, body color regulation and olfaction [31,39,40]. Previous studies in *S. gregaria* exclusively employed traditional Cas9 for editing genes other than *Crz* (e.g., *SNMP1*), while our earlier work in *L. migratoria* demonstrated the efficacy of xCas9 for *Crz* knockout [19,20]. In the present study, we successfully utilized standard Cas9 to achieve efficient *Crz* knockout in *S. gregaria*, thereby complementing existing approaches and establishing a systematic CRISPR-based editing method in desert locust. Importantly, our optimized protocols for the timing of embryo injection (limited to a 2 h window), embryo microinjection parameters (200 hPa of the injection pressure (pi), 0.5 s of the injection time (ti), and 10 hPa of the compensation pressure (pc)), and sgRNA concentration (300 ng/µL) represent a universal framework. This finding not only confirms the transferability of this technical platform within the Acrididae infraorder, but also suggests its potential broad applicability in the order Orthoptera, thereby offering significant methodological references for related research.

Our results demonstrate that compared to *L. migratoria*, *S. gregaria* G_0_ adults exhibit higher gene editing efficiency but lower survival rates. We hypothesize that this may be attributed to the relatively high injection concentrations of sgRNA/Cas9. In future studies, the optimization of injection parameters (e.g., reducing sgRNA and Cas9 dosage) could improve G_0_ survival rates, while the application of high-efficiency Cas9 variants (such as xCas9 or HiFi Cas9) may further enhance the overall editing efficiency in desert locusts. In addition, G_0_ individuals generated by CRISPR-Cas9 editing exhibited characteristic mosaicism, arising from incomplete somatic cell editing that led to the coexistence of unmodified wild-type cells and genetically modified cell populations. At the molecular level, this mosaicism was confirmed by messy peaks in Sanger sequencing chromatograms, reflecting the inherent allelic complexity of genetically mosaic organisms (Figure 3E).

In our study, phenotypic validation provided robust functional evidence of successful gene disruption, as demonstrated by the emergence of an albino phenotype in G_0_ nymphs, which served as a direct visual marker for disruption of the targeted pigmentation pathway. This visible phenotype not only confirmed editing efficacy but also facilitated rapid preliminary screening prior to molecular characterization. However, comprehensive mutational analysis at the G_0_ stage is biologically uninformative due to the critical distinction between somatic and germline mutations. Only editing events transmitted through the germline and subsequently confirmed in the G_1_ progeny represent heritable modifications. To establish stable knockout lines, we therefore performed high-resolution genotyping of G_1_/G_2_ individuals using TA cloning and deep sequencing to characterize indel profiles, followed by phenotypic correlation to identify homozygous mutant carriers. We obtained a specific mutant lacking six amino acids, where the 18-bp deletion preserved the reading frame of the downstream peptide sequence. Consistent with our findings in *L. migratoria*, although this mutation type may not represent a true null allele, it exhibited phenotypic characteristics similar to other deletion variants [19]. This observation suggests that the signal peptide plays a crucial role in generating functional Crz neuropeptides. However, further investigations are required to evaluate its potential impact and possible residual Crz activity.

The neuropeptide Crz is a key regulator of insect physiology and behavior [41,42]. In locusts, it is associated with environmental adaptation and phenotypic plasticity [19,43,44]. In *Crz* knockout experiments in *L. migratoria*, mutants exhibited an albino phenotype and reduced cuticle thickness [19]. Here, we found that *SgCrz^−/−^* mutants exhibit reduced cuticle thickness and discoloration consistent with those in *L. migratoria* [19]. This demonstrates that Crz is functionally conserved among locusts, even between genera. Likewise, gene knockdown of *Crz* with RNA interference alters cuticle color including black patterning and morphology in *S. gergaria* [30]. The *Crz* gene has high expression in the corpus allata and an albino strain lacking [His^7^]-corazonin has differences in locomotory and stationary behavior such as grooming compared to normally colored *L. migratoria* strain [30,45]. This suggests a wider, but subtle, role of Crz beyond morphology and colorization. Furthermore, our study revealed that female *SgCrz^−/−^* individuals exhibited a significantly shorter lifespan compared to wild-type.

Given the high conservation of Crz, it serves as an ideal target for gene-editing studies in locusts. Future studies could explore its role in metabolism, reproduction, and neural regulation. Edited mutants can be used to test survival or metabolic regulation under stressful conditions. *SgCrz^−/−^* mutants exhibit reduced cuticle thickness and discoloration, suggesting that Crz plays a role in the cuticle synthesis or tanning pathway. Transcriptomic analysis of mutants can identify downstream genes involved in these processes.

Visual stimulation of locusts is one of the key stimuli that trigger aggregation of locusts into devastating swarms. Therefore, in theory, CRISPR-edited *Crz* mutant locusts should reduce swarm cohesion. Disrupted integrity of the cuticle could also make the locusts more vulnerable to environmental stress [46]. Cuticle properties are optimized for flight and therefore *Crz* mutants may be limited in their range of agricultural destruction [47]. RNA interference technology can be used to develop a pest control strategy based on Crz function. Targeting *Crz* gene through RNAi can reduce melanin deposition of social locusts, weaken their group recognition and aggregation ability, and reduce the damage to pollinators and natural enemies. At the same time, it is complementary to CRISPR/Cas9 and other gene drive technologies to form a multi-level prevention and control system. It can provide a new and environmentally friendly strategy for desert locust control.

## 5. Conclusions

This study realized the application of CRISPR/Cas9 system in desert locust. We engineered mutants with an albino phenotype. Compared with wild-type locusts, there was no significant change in the phylogeny of the mutant, but the mutant cuticle was more dense. These findings not only expand the application of the CRISPR/Cas9 system in insects, but also show that the *Crz* gene is an ideal target for genome editing research in desert locusts, providing new insights into its function.

## Data Availability

The original contributions presented in this study are included in the article/Supplementary Materials. Further inquiries can be directed to the corresponding authors.

## Supplementary Materials

The following supporting information can be downloaded at: https://www.mdpi.com/article/10.3390/insects16070704/s1. Figure S1. *SgCrz* target site sequences from G_0_ egg stage.
