# Supplementary figures and images for: CRISPR/Cas9-Mediated Knockout of the Corazonin Gene Indicates Its Regulation on the Cuticle Development of Desert Locusts (Schistocerca gregaria)
Source: Insects. 2025 Jul 9;16(7):704. doi: 10.3390/insects16070704 (PMC12295317; doi:10.3390/insects16070704)

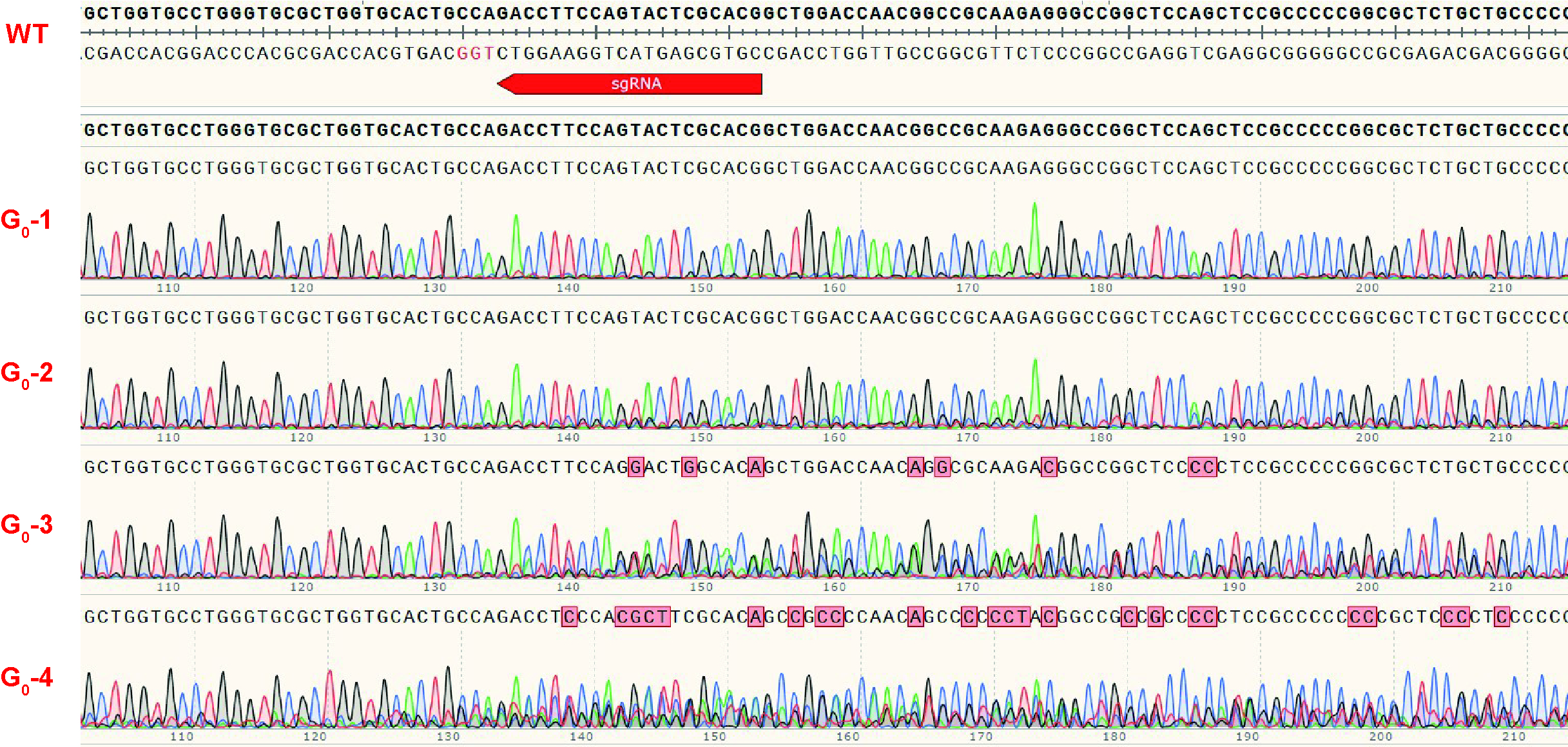

Supplement: Supplementary file 1 [file insects-16-00704-s001.zip › insects-3678017-supplementary.jpg]
